# Hospitalization Trends for Airway Infections and In-Hospital Complications in Cleft Lip and Palate

**DOI:** 10.1001/jamanetworkopen.2024.28077

**Published:** 2024-09-12

**Authors:** Rahel Laager, Claudia Gregoriano, Stephanie Hauser, Henrik Koehler, Philipp Schuetz, Beat Mueller, Alexander Kutz

**Affiliations:** 1 Department of Internal Medicine, Medical University Clinic, Kantonsspital Aarau, Aarau, Switzerland; 2 University Hospital of Child and Adolescent Psychiatry and Psychotherapy, University of Bern, Bern, Switzerland; 3 Faculty of Medicine, University of Basel, Basel, Switzerland; 4 Department of Pediatrics, Kantonsspital Graubünden, Chur, Switzerland; 5 Department of Pediatrics, Kantonsspital Aarau, Aarau, Switzerland; 6 Department of Endocrinology and Diabetology, Medical University Clinic, Kantonsspital Aarau, Aarau, Switzerland; 7 Division of Pharmacoepidemiology and Pharmacoeconomics, Department of Medicine, Brigham and Women’s Hospital and Harvard Medical School, Boston, Massachusetts

## Abstract

**Importance:**

Cleft lip or palate is a prevalent birth defect, occurring in approximately 1 to 2 per 1000 newborns and often necessitating numerous hospitalizations. Specific rates of hospitalization and complication are underexplored.

**Objective:**

To assess the rates of airway infection–associated hospitalization, overall hospital admissions, in-hospital complications, and mortality among children with a cleft lip or palate.

**Design, Setting, and Participants:**

This nationwide, population-based cohort study used in-hospital claims data from the Federal Statistical Office in Switzerland between 2012 and 2021. Participants included newborns with complete birth records born in a Swiss hospital. Data were analyzed from March to November 2023.

**Exposure:**

Prevalent diagnosis of a cleft lip or palate at birth.

**Main Outcomes and Measures:**

Outcomes of interest were monthly hospitalization rates for airway infections and any cause during the first 2 years of life in newborns with cleft lip or palate. In-hospital outcomes and mortality outcomes were also assessed, stratified by age and modality of surgical intervention.

**Results:**

Of 857 806 newborns included, 1197 (0.1%) had a cleft lip and/or palate, including 170 (14.2%) with a cleft lip only, 493 (41.2%) with a cleft palate only, and 534 (44.6%) with cleft lip and palate. Newborns with cleft lip or palate were more likely to be male (55.8% vs 51.4%), with lower birth weight (mean [SD] weight, 3135.6 [650.8] g vs 3284.7 [560.7] g) and height (mean [SD] height, 48.6 [3.8] cm vs 49.3 [3.2] cm). During the 2-year follow-up, children with a cleft lip or palate showed higher incidence rate ratios (IRRs) for hospitalizations due to airway infections (IRR, 2.33 [95% CI, 1.98-2.73]) and for any reason (IRR, 3.72 [95% CI, 3.49-3.97]) compared with controls. Additionally, children with cleft lip or palate had a substantial increase in odds of mortality (odds ratio [OR], 17.97 [95% CI, 11.84-27.29]) and various complications, including the need for intubation (OR, 2.37 [95% CI, 1.95-2.87]), extracorporeal membrane oxygenation (OR, 2.89 [95% CI, 1.81-4.63]), cardiopulmonary resuscitation (OR, 3.25 [95% CI, 2.21-4.78]), and respiratory support (OR, 1.94 [95% CI, 1.64-2.29]).

**Conclusions and Relevance:**

In this nationwide cohort study, the presence of cleft lip or palate was associated with increased hospitalization rates for respiratory infections and other causes, as well as poorer in-hospital outcomes and greater resource use.

## Key Points

**Question:**

Do children born with a cleft lip and/or palate experience a higher rate of airway infections and in-hospital complications than those without cleft lip or palate?

**Findings:**

In this cohort study of 857 806 newborns, hospitalization rates for airway infections were higher among children with a cleft lip and/or palate compared with those without. Similarly, rates of in-hospital complications and mortality were increased in children with cleft lip or palate.

**Meaning:**

These findings suggest that children with a cleft lip and/or palate had a higher risk of severe airway infections requiring hospitalization than their counterparts without a cleft.

## Teaser

This cohort study assesses rates of airway infection–associated and all-cause hospitalizations, in-hospital complications, and mortality among infants with cleft lip or palate up to age 2 years in Switzerland.

## Introduction

In Switzerland, all newborns undergo a systematic screening for lip and palate defects^1,2^ if these are not seen during prenatal ultrasonographic examination.^3,4^ Cleft lip or palate is among the most prevalent birth defects, with an incidence of approximately 1 to 2 per 1000 newborns and may be an early indication of a congenital malformation or associated syndromes.^5,6,7^ Nonetheless, 45% of cleft palates and 60% of cleft lips are diagnosed without any associated syndrome.^6,8^ Beyond feeding complications, long-term speech issues, sleep apnea, and hearing problems, cleft lip or palate has been identified as a risk factor for bronchiolitis.^9,10,11^ However, the link between these defects and other airway infections remains poorly explored. Furthermore, an association of the severity of cleft lip or palate with the need for hospitalization remains unexplored and may be an important factor for ambulatory pediatricians and family doctors to decide on a referral to the emergency department. Additionally, data on the association of corrective surgery with hospitalization risk and in-hospital resource use are scarce.^12^ The type of cleft dictates the method and timing of corrective surgery,^13,14^ with guidelines typically adhering to the Rule of 10s for lip repair: age at least 10 weeks, weight of 10 pounds, hemoglobin greater than 10 g/dL (to convert to grams per liter, multiply by 10), and white blood cell count exceeding 10 000 μL (to convert to ×10^9^/L, multiply by 0.001).^15,16^ However, this rule does not account for the overall disease burden of cleft lip or palate and associated risks. Since hospitalizations for newborns or young children with cleft lip or palate typically require urgent care and a thorough interdisciplinary in-hospital work-up to prevent severe complications, understanding epidemiological trends and potential in-hospital adverse outcomes is therefore crucial.^17^ Hence, in this nationwide cohort study, our primary goal was to examine the incidence rates of hospitalizations due to respiratory infections or any cause during the first 2 years of life, considering the necessity and timing of corrective surgery. Our secondary aim was to assess clinical outcomes of newborns and young children with cleft lip or palate compared with those without clefts. Thus, this analysis aims to enhance our comprehension of the disease burden among hospitalized patients with cleft lip or palate.

## Methods

This cohort study received a waiver of the need for an ethical authorization and informed consent from the institutional review board of Northwestern and Central Switzerland due to the use of exclusively anonymous data. This study adheres to the Strengthening the Reporting of Observational Studies in Epidemiology (STROBE) reporting guideline.

### Study Design

This analysis was conducted using a nationwide cohort of hospitalized patients with cleft lip or palate in Switzerland between 2012 and 2021. Hospitalization data were provided by the Swiss Federal Statistical Office, based on nationwide compulsory full census of Swiss hospitals. The dataset includes all Swiss inpatient discharge records from acute care, general, and specialty hospitals for both pediatric and adult patients. Individual-level data on patient demographics, health care utilization, hospital typology, medical diagnoses, diagnostic tests, clinical procedures, and in-hospital patient outcomes were provided. Multistep anonymization procedure ensured patient confidentiality, and a unique patient identifier was used to ascertain rehospitalizations. Medical diagnoses were coded using the *International Statistical Classification Of Diseases And Related Health Problems, 10th revision, German Modification* (*ICD-10-GM*) codes, while interventions, like surgical cleft repairs, were coded using Swiss classification of surgical interventions (CHOP) codes.

### Case Ascertainment and Study Variables

In this analysis, we included data for all children born in Swiss hospitals from 2012 to 2021, ensuring they had complete birth records and tracking any rehospitalizations within their first 2 years of life. Children with a cleft lip and/or palate were identified using the *ICD-10-GM* codes Q35 to Q37.^18^ We excluded newborns diagnosed with other congenital malformations of the tongue, mouth, and pharynx (*ICD-10-GM* code Q38), if they did not have a diagnosed cleft lip or palate.

Baseline characteristics, such as comorbidities, birth complications, and details of cleft repair interventions, were determined through *ICD-10-GM* and CHOP codes. A comprehensive list of all *ICD-10-GM* and CHOP codes used in this study is provided in eTable 1 in Supplement 1.

### Outcomes

The primary outcome of this study was the rate of hospitalizations due to airway infections. Secondary outcomes included hospitalization rates for any cause after birth, all-cause mortality, hospital length of stay (LOS), and hospitalization-associated complications. These complications were defined as the need for respiratory support, extracorporeal membrane oxygenation, resuscitation, intubation, admission to the intensive care unit, or in-hospital mortality. Hospitalizations specifically for cleft repair were excluded from the analysis because postsurgical care may often include respiratory support, intensive care unit stay, and intubation.

### Statistical Analysis

We calculated baseline characteristics of the eligible newborns using analysis of variance or Pearson χ^2^ test and indicated them as mean and SD or number and percentage. We determined the distribution of patients according to the modality of corrective surgery on a monthly basis for the first 2 years of life. Initially, all newborns were categorized as either control (without cleft) or cleft without surgery and were reassessed monthly. Patients underwent reclassification into the lip repair group following their lip repair surgery and were later moved to the palate repair group in case they underwent any subsequent palate repair, regardless of any previous lip repair.

To visualize the incidence rates of hospitalization due to respiratory infection or any cause, we used locally weighted scatterplot smoothing, stratified by the modality of corrective surgery performed. The age at first surgery was displayed as a smoothed histogram, and the difference in distribution was assessed using a Kolmogorov-Smirnov test.

To assess potential differences in outcomes associated with corrective surgery, we defined 2 timeframes (before and after surgery) for patients with a cleft lip or palate and a documented corrective surgery. Hospitalizations from controls were allocated to a timeframe based on their age in reference to the mean age at which patients with clefts underwent their initial surgery. Controls who died before reaching the mean age of surgery were not included in the postsurgical cohort. For both the presurgical and postsurgical periods, we calculated incidence rate ratios (IRRs) using Poisson regression models to compare incidence rates between individuals with and without clefts. Differences in in-hospital complications and resource use were analyzed using logistic and linear regression models. Subgroup analyses were specifically conducted for cleft lip repair only and cleft palate repair only. To test the robustness of our findings, a sensitivity analysis was performed comparing hospitalizations involving airway infections at any position (primary and secondary diagnosis) vs those where it was at the primary position (primary diagnosis only).

All *P* values are 2-sided and have not been adjusted for multiple testing. Results were considered statistically significant at *P* < .05. All statistical analyses were executed with Stata software version 17.0 (StataCorp). Data were analyzed from Data were analyzed from March to November 2023.

## Results

### Baseline Characteristics

**Figure 1.**
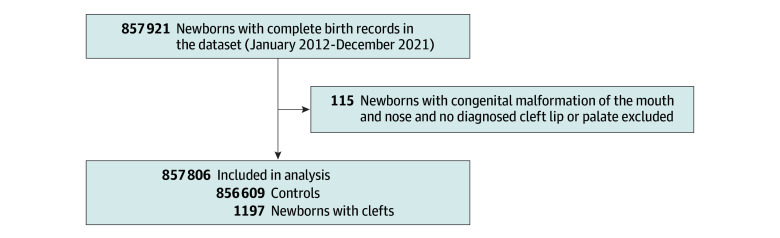
Study Enrollment Flowchart

Between January 2012 and December 2021, the complete birth records included 857 806 children born in a Swiss hospital (Figure 1), which is 99.6% of all births in Swiss hospitals during the study period.^19^ Among these, 1197 children (0.1%) were identified with a diagnosis of cleft lip or palate, including 170 children (14.2%) with a cleft lip only, 495 children (41.2%) with a cleft palate only, and 534 children (44.6%) with both cleft lip and palate. Newborns with a cleft were more likely to be male (55.8% vs 51.4%), with lower birth weight (mean [SD] weight, 3135.6 [650.8] g vs 3284.7 [560.7] g) and height (mean [SD] height, 48.6 [3.8] cm vs 49.3 [3.2] cm). Children with a cleft were more often diagnosed with other types of congenital malformations and syndromes (34.2% vs 5.8%) and experienced more complications during or after birth than those in the control group. For instance, respiratory issues at birth were observed in 10.8% of children with cleft, compared with 2.9% of children in the control group; adaptive dysfunction was noted in 6.3% vs 2.3%; and the proportion of mortality during birth hospitalization was 1.8%, compared with 0.6% in the control group (Table). Baseline characteristics stratified by type of cleft are shown in eTable 2 in Supplement 1.

**Table.** Baseline Characteristics

Characteristic	Newborns, No. (%)			*P* value	Standardized difference
	Total (N = 857 806)	Controls (n = 856 609)	Clefts (n = 1197)		
Sex					
Female	416 962 (48.6)	416 433 (48.6)	529 (44.2)	.002	0.09
Male	440 844 (51.4)	440 176 (51.4)	668 (55.8)		
Types of clefts					
Cleft palate only	493 (0.1)	NA	493 (41.2)	<.001	3.48
Cleft lip only	170 (<0.1)	NA	170 (14.2)		
Cleft palate with cleft lip	534 (0.1)	NA	534 (44.6)		
Birth characteristics					
Multiple birth					
1	827 305 (96.4)	826 146 (96.4)	1159 (96.8)	.78	0.02
2	29 665 (3.5)	29 628 (3.5)	37 (3.1)		
≥3	836 (0.1)	835 (0.1)	1 (0.1)		
Birth weight, mean (SD), g	3284.5 (560.9)	3284.7 (560.7)	3135.6 (650.8)	<.001	0.25
Birth height, mean (SD), cm	49.3 (3.2)	49.3 (3.2)	48.6 (3.8)	<.001	0.22
Gestational age, mean (SD), wk	39.1 (2.0)	39.1 (2.0)	38.9 (2.1)	<.001	0.13
Congenital diseases and malformations					
Chromosomal anomalies	1335 (0.2)	1294 (0.2)	41 (3.4)	<.001	−0.25
Congenital malformations of the respiratory system	1660 (0.2)	1584 (0.2)	76 (6.3)	<.001	−0.35
Cystic fibrosis	142 (<0.1)	142 (<0.1)	0	.66	0.02
Congenital malformations of the heart	8057 (0.9)	7918 (0.9)	139 (11.6)	<.001	−0.45
Congenital malformations of the vascular system	5338 (0.6)	5267 (0.6)	71 (5.9)	<.001	−0.30
Other congenital malformations of the digestive system	7617 (0.9)	7527 (0.9)	90 (7.5)	<.001	−0.34
Metabolic disorders	360 (<0.1)	357 (<0.1)	3 (0.3)	<.001	−0.05
Congenital malformations of the urinary system	6025 (0.7)	5971 (0.7)	54 (4.5)	<.001	−0.24
Congenital malformations of genital organs	7294 (0.9)	7236 (0.8)	58 (4.8)	<.001	−0.24
Congenital malformations and deformations of the musculoskeletal system	17 748 (2.1)	17 573 (2.1)	175 (14.6)	<.001	−0.47
Other congenital malformations	4692 (0.5)	4503 (0.5)	189 (15.8)	<.001	−0.58
Congenital syndromes combined	49 963 (5.8)	49 554 (5.8)	409 (34.2)	<.001	−0.76
Neonatological issues					
Intrauterine hypoxia	22 823 (2.7)	22 770 (2.7)	53 (4.4)	<.001	−0.10
Birth asphyxia	17 333 (2.0)	17 291 (2.0)	42 (3.5)	<.001	−0.09
Adaptive dysfunction	19 467 (2.3)	19 391 (2.3)	76 (6.3)	<.001	−0.20
Neonatal respiratory issues	25 389 (3.0)	25 260 (2.9)	129 (10.8)	<.001	−0.31
Respiratory distress of newborn	65 805 (7.7)	65 583 (7.7)	222 (18.5)	<.001	−0.33
Neonatal cardiovascular issues	20 185 (2.4)	20 086 (2.3)	99 (8.3)	<.001	−0.27
Neonatal hemorrhages	4697 (0.5)	4681 (0.5)	16 (1.3)	<.001	−0.08
Neonatal hemolysis	5006 (0.6)	4999 (0.6)	7 (0.6)	>.99	0.00
Other perinatal hematological disorders	9767 (1.1)	9720 (1.1)	47 (3.9)	<.001	−0.18
Neonatal transitory endocrine and metabolic disorders	27 724 (3.2)	27 620 (3.2)	104 (8.7)	<.001	−0.23
Neonatal digestive system disorders	3970 (0.5)	3937 (0.5)	33 (2.8)	<.001	−0.18
Neonatal temperature regulation and integument conditions	25 929 (3.0)	25 888 (3.0)	41 (3.4)	.42	−0.02
Neurological disorders of fetus and newborn	4903 (0.6)	4858 (0.6)	45 (3.8)	<.001	−0.22
Death in birth hospitalization	5033 (0.6)	5011 (0.6)	22 (1.8)	<.001	−0.11
Resuscitation	21 512 (2.5)	21 431 (2.5)	81 (6.8)	<.001	−0.20
Intubation	16 243 (1.9)	16 163 (1.9)	80 (6.7)	<.001	−0.24

Abbreviation: NA, not applicable.

### Surgical Interventions

**Figure 2.**
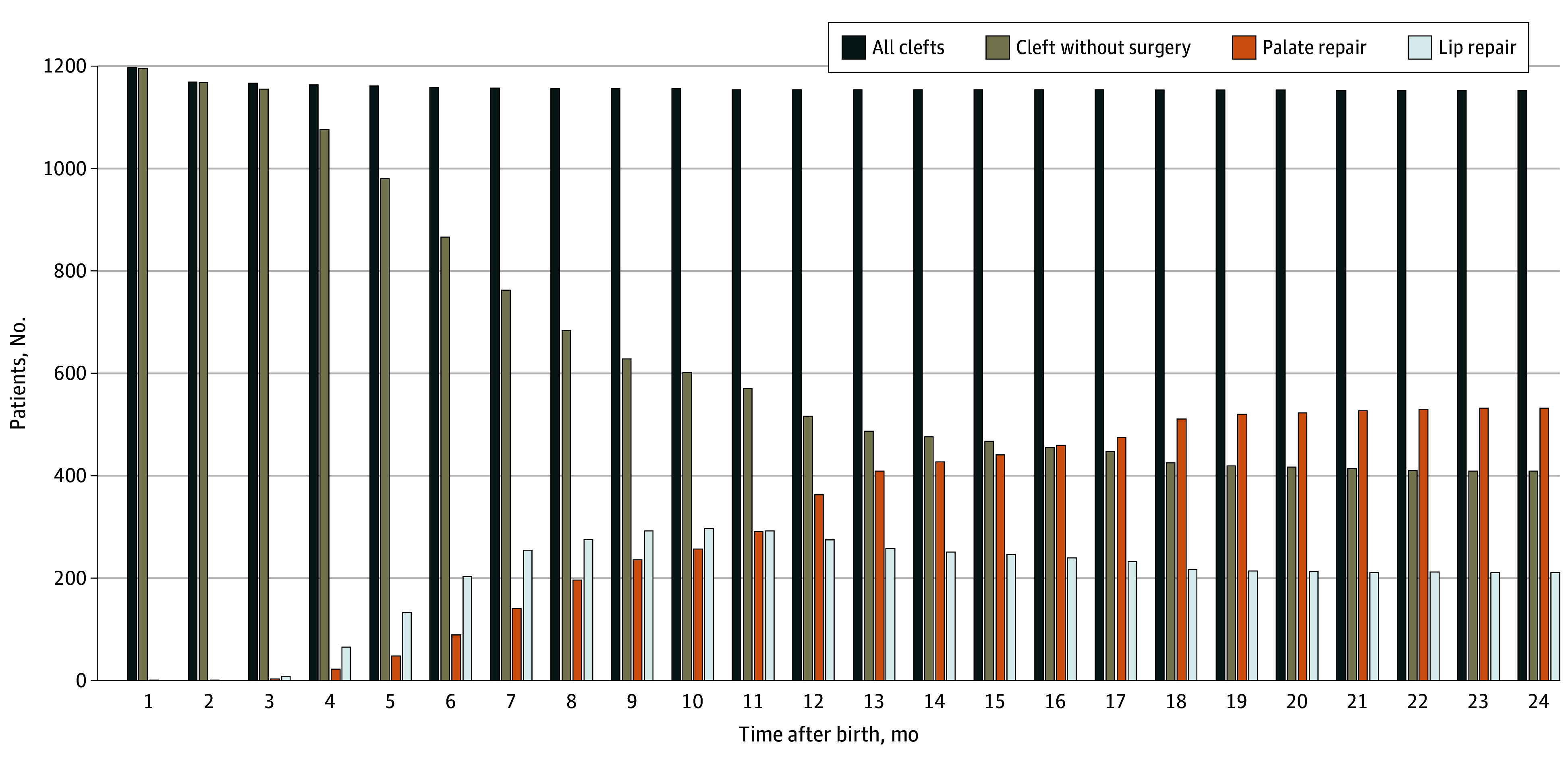
Distribution of Surgical Cleft Repair by Age

Within the first 2 years of life, 744 children with a cleft (62.2%) underwent a cleft repair surgery. Of those, 211 children (28.4%) had a lip repair only, 291 children (39.1%) had a palate repair only, and 242 children (32.5%) underwent both lip and palate repair surgery (Figure 2; eTable 3 in Supplement 1). Characteristics at birth for children who did not receive cleft repair surgery are shown in eTable 3 in Supplement 1. The most common cleft repair procedures were primary plastic reconstruction on the soft palate (417 children) and cheiloplasty for a congenital cleft lip (398 children) (eFigure 1 in Supplement 1). The mean age at the time of the first surgery was 236 (95% CI, 226.94-244.71) days for any corrective surgery, 313 (95% CI, 300.96-324.72) days for palate repair, and 181 (95% CI, 174.24-187.82) days for lip repair, with significant differences in distribution observed between the types of surgery (*P* < .001) (eFigure 2 in Supplement 1).

### Hospitalizations Due to Airway Infections or Any Cause

**Figure 3.**
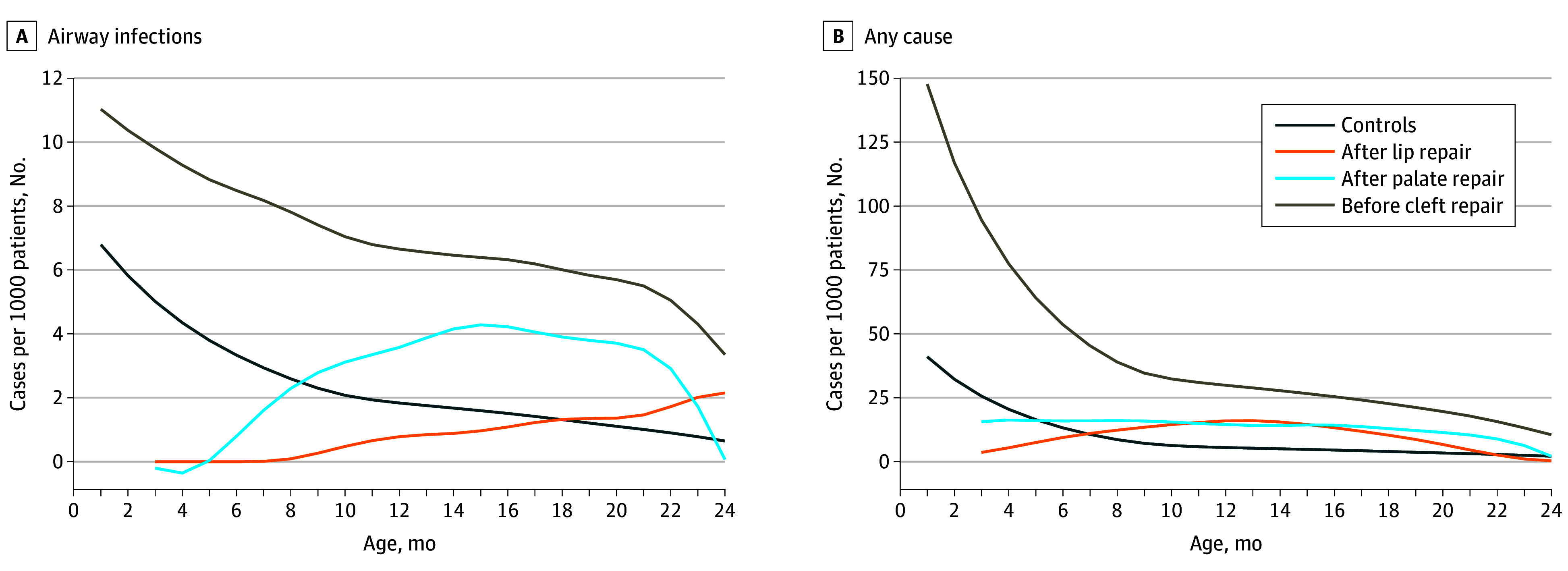
Incidence Rates of Hospitalizations Due to Airway Infections or Any Cause by Age

During the 2-year follow-up, hospitalization rates for airway infections (IRR, 2.33 [95% CI, 1.98-2.73]) and for any cause (IRR, 3.72 [95% CI, 3.49-3.97]) were higher in children with a cleft lip or palate compared with controls. The highest rates for both outcomes were observed in children with uncorrected clefts, followed by those who had undergone palate repair, lip repair, and lastly, controls (Figure 3). Incidence rates for airway infections were higher for children before cleft repair surgery (IRR, 2.38 [95% CI, 1.82-3.11]) compared with after surgery (IRR, 1.87 [95% CI, 1.33-2.64]). However, incidence rates for hospitalizations due to any cause were lower before cleft repair (IRR, 4.06 [95% CI, 3.68 to 4.47]) compared with after surgery (IRR, 6.55 [95% CI, 5.90 to 7.28]) compared with children without a cleft (eFigure 3 in Supplement 1).

### In-Hospital Outcomes

**Figure 4.**
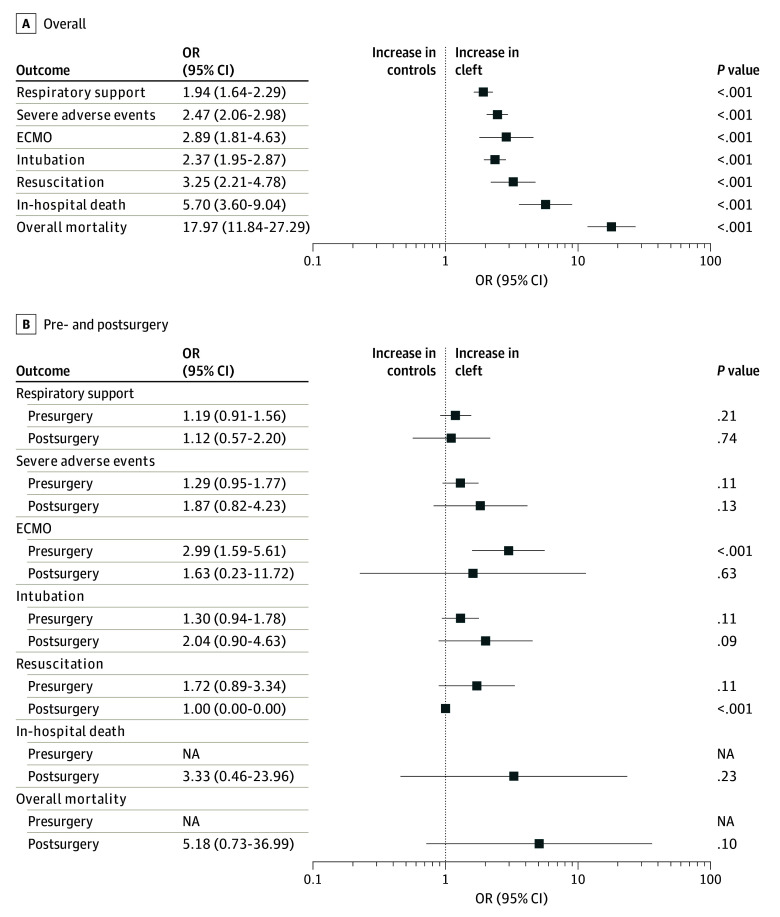
In-Hospital Outcomes and Mortality ECMO indicates extracorporeal membrane oxygenation; NA, not applicable; OR, odds ratio.

Compared with children without a cleft lip or palate, those born with these conditions had increased odds of in-hospital complications and higher resource use. Moreover, these patients experienced relatively longer hospital LOS for airway infections (β = 2.25 [95% CI, 1.71-2.78]) and for any causes of hospitalization (β = 5.75 [95% CI, 5.04-6.46]) than their counterparts without any clefts. Analyzing the data by classifying patients into before and after cleft repair surgery groups, the trends remained similar in the presurgery group, except for the need for resuscitation. In contrast, in the postsurgery group, there were no significant differences in odds compared with the control group (Figure 4).

### Subgroup and Sensitivity Analyses

Patients with a planned cleft lip repair had a notable increase in the incident rate for hospitalizations due to airway infections prior to surgery compared with controls (IRR, 1.83 [95% CI, 1.20-2.81]). However, after surgery, the incidence rate did not significantly differ from that of the control group (IRR, 1.17 [95% CI, 0.70-1.98]). Conversely, the incidence rate for hospitalizations of any cause significantly increased after lip repair (IRR, 6.78 [95% CI, 6.00-7.67]) compared with the presurgery group (IRR, 3.42 [95% CI, 2.97-3.94]) (eFigure 4 in Supplement 1). When compared with controls, patients with a cleft lip showed no significant difference in the odds of requiring intubation, resuscitation, or respiratory support, either before or after lip repair surgery (eFigure 5 in Supplement 1).

Children with a cleft palate showed a higher incidence rate for hospitalizations due to airway infections both before (IRR, 2.56 [95% CI, 1.92-3.43]) and after (IRR, 2.93 [95% CI, 2.02-4.24]) palate repair surgery compared with controls. The incident rate for hospitalizations for any cause were also higher before (IRR, 6.19 [95% CI, 5.67-6.76]) and after (IRR, 5.47 [ 95% CI, 4.69-6.37]) surgery compared with controls (eFigure 6 in Supplement 1). Before undergoing cleft palate repair, patients showed increased odds for in-hospital complications compared with controls, including the need for intubation (odds ratio [OR], 2.37 [95% CI, 1.95-2.87]), extracorporeal membrane oxygenation (OR, 2.89 [95% CI, 1.81-4.63]), cardiopulmonary resuscitation (OR, 3.25 [95% CI, 2.21-4.78]), and respiratory support (OR, 1.94 [95% CI, 1.64-2.29]). Furthermore, children with cleft lip or palate had a substantial increase in odds of mortality (OR, 17.97 [95% CI, 11.84-27.29]). This difference in odds was no longer significant after the corrective surgery (eFigure 7 in Supplement 1). In a sensitivity analysis considering hospitalizations with any diagnosis of airway infection (primary or secondary diagnosis), the results remained consistent (eTable 4 and eFigure 8 in Supplement 1).

## Discussion

In this cohort study of 857 806 newborns in Swiss hospitals from 2012 to 2021, 3 key findings emerged. First, children with cleft lip or palate were more likely to have concomitant congenital issues and birth complications. Second, a significant proportion of these children received cleft repair surgery, primarily soft palate reconstruction and cheiloplasty for cleft lips, within the first 2 years of life. Third, these children exhibited higher hospitalization rates for airway infections and other causes, both before and after surgery. Additionally, compared with children without these conditions, children with cleft lip or palate had increased risks of in-hospital complications and mortality, and greater resource use.

Our study highlights that children born with a cleft lip or palate had a higher relative risk of hospitalization due to airway infections, supporting the findings from a 2020 analysis that identified cleft lip or palate as a risk factor for bronchiolitis.^10^ In our cohort, children with a cleft lip or palate who had not yet undergone corrective surgery were particularly prone to hospitalizations for airway infections. This risk may be linked to a higher prevalence of congenital malformations and neonatological complications, often rendering these children ineligible for early surgery according to the historical Rule of 10s.^15,16^ Therefore, it is plausible to suggest a reversed association: the severity of the patients’ conditions and their propensity for hospitalizations might delay their eligibility for corrective surgery within the first 2 years of life.

Nonetheless, children who underwent corrective surgery within this timeframe continued to have a higher risk of airway infection–related hospitalizations, especially before the cleft repair, although this risk persisted to a certain extent after surgery as well. Notably, when stratifying between surgical modalities, the increased hospitalization rates for airway infections remained high after palate repair but normalized after lip repair. This aligns with the varying severity of cleft types and their corresponding surgical interventions.^17^

Regarding the overall increased risk of a rehospitalization, it is plausible to suggest that patients with a cleft lip or palate may not necessarily experience more severe airway infections but are more likely to be hospitalized due to increased vigilance by parents and health care practitioners.^20^ This greater awareness could be linked to feeding difficulties observed in these children, which is consistent with reduced fluid intake as a criterion for hospitalization in children with airway infections.^21,22,23^ However, our study also identified an elevated relative risk for in-hospital complications, greater resource utilization, higher mortality, indicating that these patients indeed suffer from more severe illness.^12,24,25^

While we observed decreasing hospitalization rates for airway infections after corrective surgery, the hospitalization rates for any cause were increased compared with controls even after corrective surgery. This phenomenon could be explained by the fact that the first corrective surgery is used as a reference point, especially since nearly half of our population had both a cleft lip and palate, necessitating multiple corrective procedures. Moreover, given that the presence of a cleft lip or palate often coexists with a larger syndrome, the elevated hospitalization rates may be attributed to a higher prevalence of comorbidities.^6,8,23^ These patients also experienced a longer hospital LOS, further supporting the notion that their medical condition was more severe. Importantly, the risk of airway infection, complications, and hospital LOS all showed a reduction after corrective surgery.

The optimal timing for cleft repair surgery has been debated for years.^14,26,27^ Findings reported by Gamble et al^13^ in 2023 have shown that corrective surgery performed at age 6 months is safer and yields better outcomes for velopharyngeal insufficiency compared with surgery at age 12 months. In line with our results, this evidence further supports the benefits of an earlier surgical correction.

Within our cohort, only 62.2% of infants with a cleft underwent a cleft repair within the first 2 years of their lives. Factors for a delayed surgical intervention have been a focus of study for years. While most research has focused on ethical and socioeconomic factors, the baseline characteristics for the infants not receiving any cleft repair surgery in this cohort suggest birth complications and comorbidities were relevant factors in delayed or missing cleft repair.^13,28,29,30,31,32^

Our analysis has several strengths, with the most significant being the large patient cohort and near-comprehensive nationwide records. This is particularly bolstered by the fact that newborn assessments in hospitals routinely include lip and palate examinations, thus leading to a high detection rate during birth hospitalizations, which can be assumed to closely approximate the actual prevalence.^2^ Consequently, our findings demonstrate high external validity, particularly among countries sharing a similar economic status, health care system, and genetic makeup of the population. Furthermore, this study represents the first of its kind, focusing on hospitalizations due to airway infections in patients with a cleft lip or palate within such a comprehensive study population.

### Limitations

This study has limitations. Since the *ICD-10-GM* codes were primarily collected for billing purposes, underreporting and misclassification are likely. Nonetheless, the high incidence rate of cleft lip or palate in our study population, along with evidence from previous studies, suggests that identification rates are likely high.^5,18^ Unfortunately, we were not able to stratify by syndromic vs nonsyndromic cleft due to the lack *ICD-10-GM* codes for rare syndromes. Therefore, some hospitalizations in the cleft group may be due to other syndromic reasons, leading to a slightly overestimated hospitalization rate. Our study’s reliance on hospital claims data introduces certain limitations, particularly in assessing outpatient health care utilization. Therefore, oronasal fistulae and growth or speech issues could not be assessed. However, we were able to include data on out-of-hospital mortality. Additionally, a loss to follow up, such as moving away from Switzerland, cannot be assessed, leading to a slightly lower hospitalization rate. This likely would be equally present in both groups and therefore not compromise the results. Due to the availability of solely admission dates, we had to use age at admission as a proxy for age at surgery, leading to a slightly younger mean age at the time of surgery. While we possessed age data in days for the first year, we had to estimate ages beyond 12 months by considering the month of admission and birth. Since these limitations affect both the controls and children with clefts, we do not assume a relevant bias.

## Conclusions

In this cohort study including 857 806 newborns, we observed an association of the presence of cleft lip and/or palate with higher hospitalization rates for airway infections and all-cause rehospitalizations. This association was particularly pronounced among individuals who had not undergone corrective surgery. Furthermore, children with cleft lip or palate exhibited more unfavorable in-hospital outcomes and greater resource utilization compared with their counterparts without these conditions.

## Notes

Supplement 1. **eTable 1**. *ICD-10-GM* and CHOP Codes **eTable 2.** Baseline Characteristics by Cleft Type **eTable 3.** Baseline Characteristics With or Without Surgical Cleft Repair **eTable 4.** Incidence Rate Ratios (IRR) for Hospitalizations Considering Any Diagnosis of Airway Infection (Primary or Secondary Diagnosis) **eFigure 1.** Distribution of Surgical Modalities **eFigure 2.** Distribution of Surgical Cleft Repair by Age **eFigure 3.** Hospitalization Rates Before and After First Cleft Repair **eFigure 4.** Hospitalization Rates Before and After First Lip Repair **eFigure 5.** In-Hospital Outcomes Before and After Surgical Lip Repair **eFigure 6.** Hospitalization Rates Before and After First Palate Repair **eFigure 7.** In-Hospital Outcomes Before and After Surgical Palate Repair **eFigure 8.** Monthly Incidence Rates of Hospitalizations Considering Any Diagnosis of Airway Infection (Primary or Secondary Diagnosis)

Supplement 2.
Data Sharing Statement
