# Supplementary material for: Hospitalization Trends for Airway Infections and In-Hospital Complications in Cleft Lip and Palate
Source: JAMA Netw Open. 2024 Sep 12;7(9):e2428077. doi: 10.1001/jamanetworkopen.2024.28077 (PMC11393727; doi:10.1001/jamanetworkopen.2024.28077)
Supplement: Supplement 1. — eTable 1. ICD-10-GM and CHOP Codes eTable 2. Baseline Characteristics by Cleft Type eTable 3. Baseline Characteristics With or Without Surgical Cleft Repair eTable 4. Incidence Rate Ratios (IRR) for Hospitalizations Considering Any Diagnosis of Airway Infection (Primary or Secondary Diagnosis) eFigure 1. Distribution of Surgical Modalities eFigure 2. Distribution of Surgical Cleft Repair by Age eFigure 3. Hospitalization Rates Before and After First Cleft Repair eFigure 4. Hospitalization Rates Before and After First Lip Repair eFigure 5. In-Hospital Outcomes Before and After Surgical Lip Repair eFigure 6. Hospitalization Rates Before and After First Palate Repair eFigure 7. In-Hospital Outcomes Before and After Surgical Palate Repair eFigure 8. Monthly Incidence Rates of Hospitalizations Considering Any Diagnosis of Airway Infection (Primary or Secondary Diagnosis) [file jamanetwopen-e2428077-s001.pdf]

Supplemental Online Content

Laager R, Gregoriano C, Hauser S, et al. Hospitalization trends for airway infections and in-hospital complications in cleft lip and palate. *JAMA Netw Open*. 2024;7(9):e2428077. doi:10.1001/jamanetworkopen.2024.28077

eTable 1. *ICD-10-GM* and CHOP Codes
eTable 2. Baseline Characteristics by Cleft Type
eTable 3. Baseline Characteristics With or Without Surgical Cleft Repair
eTable 4. Incidence Rate Ratios (IRR) for Hospitalizations Considering Any Diagnosis of Airway Infection (Primary or Secondary Diagnosis)
eFigure 1. Distribution of Surgical Modalities
eFigure 2. Distribution of Surgical Cleft Repair by Age
eFigure 3. Hospitalization Rates Before and After First Cleft Repair
eFigure 4. Hospitalization Rates Before and After First Lip Repair
eFigure 5. In-Hospital Outcomes Before and After Surgical Lip Repair
eFigure 6. Hospitalization Rates Before and After First Palate Repair
eFigure 7. In-Hospital Outcomes Before and After Surgical Palate Repair
eFigure 8. Monthly Incidence Rates of Hospitalizations Considering Any Diagnosis of Airway Infection (Primary or Secondary Diagnosis)

This supplemental material has been provided by the authors to give readers additional information about their work.

© 2024 Laager R et al. *JAMA Network Open.*

Table S1 – ICD & CHOP-Codes

a. Cohort definition		
**ICD-10/CHOP**	**Code**	**Definition**
**ICD-10**	**Q35.-**	**Cleft palate**
ICD-10	Q35.1	Cleft hard palate
ICD-10	Q35.3	Cleft soft palate
ICD-10	Q35.5	Cleft hard palate with cleft soft palate
ICD-10	Q35.7	Cleft uvula
ICD-10	Q35.9	Cleft palate, unspecified
**ICD-10**	**Q36.-**	**Cleft lip**
ICD-10	Q36.0	Cleft lip, bilateral
ICD-10	Q36.1	Cleft lip, median
ICD-10	Q36.9	Cleft lip, unilateral
**ICD-10**	**Q37.-**	**Cleft palate with cleft lip**
ICD-10	Q37.0	Cleft hard palate with bilateral cleft lip
ICD-10	Q37.1	Cleft hard palate with unilateral cleft lip
ICD-10	Q37.2	Cleft soft palate with bilateral cleft lip
ICD-10	Q37.3	Cleft soft palate with unilateral cleft lip
ICD-10	Q37.4	Cleft hard and soft palate with bilateral cleft lip
ICD-10	Q37.5	Cleft hard and soft palate with unilateral cleft lip
ICD-10	Q37.8	Unspecified cleft palate with bilateral cleft lip
ICD-10	Q37.9	Unspecified cleft palate with unilateral cleft lip
**ICD-10**	Q38.-	**Other congenital malformations of tongue, mouth and pharynx**

© 2024 Laager R et al. *JAMA Network Open.*

b. Interventions		
**ICD-10/CHOP**	**Code**	**Definition**
**CHOP**	**27.54**	**Plastic reconstruction of a (congenital) cleft lip**
CHOP	27.54.00	Plastic reconstruction of a (congenital) cleft lip, unspecified
CHOP	27.54.10	Cheiloplasty of a (congenital) cleft lip
CHOP	27.54.11	Secondary surgery of a (congenital) cleft lip
CHOP	27.54.99	Plastic reconstruction of a (congenital) cleft lip, other
**CHOP**	**27.62**	**Plastic reconstruction of a cleft palate**
CHOP	27.62.10	Primary plastic reconstruction on the bony palate without bone graft
CHOP	27.62.11	Primary plastic reconstruction on the bony palate without bone graft
CHOP	27.62.12	Primary plastic reconstruction on the soft palate
CHOP	27.62.20	Secondary plastic reconstruction on the bony palate without bone graft
CHOP	27.62.21	Secondary plastic reconstruction on the bony palate with bone graft
CHOP	27.62.22	Secondary plastic reconstruction on the soft palate
CHOP	27.62.99	Correction of cleft palate, other
**CHOP**	27.64	**Insertion of a palatal implant**
**CHOP**	27.63	**Revision of the correction of a cleft palate**
**CHOP**	27.69	**Other plastic reconstruction of the palate**

© 2024 Laager R et al. *JAMA Network Open.*

c. Outcomes		
**ICD-10/CHOP**	**Code**	**Definition**
**Hospitalization due to airway infection**		
ICD-10	A37.-	Whooping cough
ICD-10	A48.1	Legionnaires disease
ICD-10	J00.-	Acute nasopharyngitis [common cold]
ICD-10	J01.-	Acute sinusitis
ICD-10	J02.-	Acute pharyngitis
ICD-10	J03.-	Acute tonsillitis
ICD-10	J04.-	Acute laryngitis and tracheitis
ICD-10	J05.-	Acute obstructive laryngitis [croup] and epiglottitis
ICD-10	J06.-	Acute upper respiratory infections of multiple and unspecified sites
ICD-10	J10.-	Influenza due to identified seasonal influenza virus
ICD-10	J11.-	Influenza, virus not identified
ICD-10	J12.-	Viral pneumonia, not elsewhere classified
ICD-10	J13.-	Pneumonia due to Streptococcus pneumoniae
ICD-10	J14.-	Pneumonia due to Haemophilus influenzae
ICD-10	J15.-	Bacterial pneumonia, not elsewhere classified
ICD-10	J16.-	Pneumonia due to other infectious organisms, not elsewhere classified
ICD-10	J17.-	Pneumonia in diseases classified elsewhere
ICD-10	J18.-	Pneumonia, organism unspecified
ICD-10	J20.-	Acute bronchitis
ICD-10	J21.-	Acute bronchiolitis
ICD-10	J22.-	Unspecified acute lower respiratory infection
ICD-10	J85.-	Abscess of lung and mediastinum
**Resuscitation**		
CHOP	93.93.-, 99.6.-	Non-mechanical methods of resuscitation, Conversion of the cardiac rhythm,

© 2024 Laager R et al. *JAMA Network Open.*

ICD-10/CHOP	Code	Definition
**Respiratory support**		
CHOP	93.96.-	Oxygen enrichment
CHOP	93.9C.11, 93.90.1	Continuous positive airway pressure
CHOP	93.9E.-, 93.90.-	Non-invasive ventilation
CHOP	93.9F.-, 96.A1.-	Mechanical ventilation and respiratory support
CHOP	93.9G.-	Treatment of respiratory regulation disorders outside the intensive care unit
CHOP	93.91.-	Intermittent positive pressure breathing
**Extracorporeal membrane oxygenation**		
CHOP	37.5.-	Cardiac replacement procedures
CHOP	37.6.-	Implantation of cardiac and circulatory support systems
CHOP	39.61.-	Extracorporeal circulation
CHOP	39.65.-	Extracorporeal membrane oxygenation [ECMO] and pre-ECMO therapy
CHOP	37.9A.-	Implantation of prosthetic ventricular assist device on the heart
CHOP	37.41.-	Implantation of prosthetic ventricular assist device on the heart

© 2024 Laager R et al. *JAMA Network Open.*

d. baseline characteristics		
**ICD-10/CHOP**	**Code**	**Definition**
**Congenital diseases and malformations**		
ICD-10	Q90, Q91, Q92, Q93, Q95, Q96, Q97, Q98, Q99, E791	Chromosomal anomalies
ICD-10	Q30, Q31, Q32, Q33, Q34	Congenital malformations of the respiratory system
ICD-10	E84	Cystic fibrosis
ICD-10	Q20, Q21, Q22, Q23, Q24	Congenital malformations of the heart
ICD-10	Q25, Q26, Q27, Q28	Congenital malformations of the vascular system
ICD-10	Q38, Q39, Q40, Q41, Q42, Q43, Q44, Q45	Other congenital malformations of the digestive system
ICD-10	E70, E71, E72, E74, E75, E76, E77	Metabolic disorders
ICD-10	Q60, Q61, Q62, Q63, Q64	Congenital malformations of the urinary system
ICD-10	Q50, Q51, Q52, Q53, Q54, Q55, Q56	Congenital malformations of genital organs
ICD-10	Q65, Q66, Q67, Q68, Q69, Q70, Q71, Q72, Q73, Q74, Q75, Q76, Q77, Q78, Q79	Congenital malformations and deformations of the musculoskeletal system
ICD-10	Q80, Q81, Q82, Q83, Q84, Q85, Q86, Q87, Q88, Q89	Other congenital malformations

© 2024 Laager R et al. *JAMA Network Open.*

ICD-10/CHOP	Code	Definition
**Neonatological issues**		
CHOP	99.65.-	Adaptive dysfunction
ICD-10	P20	Intrauterine hypoxia
ICD-10	P21	Birth asphyxia
ICD-10	P23, P24, P25, P26, P27, P28	Neonatal respiratory issues
ICD-10	P22, P285	Respiratory distress of newborn
ICD-10	P29	Neonatal cardiovaskular issues
ICD-10	P50, P51, P52, P53, P54	Neonatal haemorrhages
ICD-10	P55, P56, P57, P58	Neonatal haemolysis
ICD-10	P60, P61	Other perinatal haematological disorders
ICD-10	P70, P71, P72, P73, P74	Neonatal transitory endocrine & metabolic disorders
ICD-10	P75, P76, P77, P78	Neonatal digestive system disorders
ICD-10	P80, P81, P83	Neonatal temperature regulation and integument conditions
ICD-10	P90, P91, P94	Neurological disorders of fetus and newborn

© 2024 Laager R et al. *JAMA Network Open.*

Table S2 – Baseline characteristics by cleft type

Characteristic	Cleft palate only, n (%)	Cleft lip only, n (%)	Cleft lip and palate, n (%)	P-value
**N**	N=493	N=170	N=534	
**Sex**				<0.001
Female	270 (54.8)	60 (35.3)	199 (37.3)	
Male	223 (45.2)	110 (64.7)	335 (62.7)	
**Type of surgery**				<0.001
None	242 (49.1)	62 (36.5)	149 (27.9)	
Lip repair only	0 (0.0)	108 (63.5)	103 (19.3)	
Palate repair only	251 (50.9)	0 (0.0)	40 (7.5)	
Lip and palate repair	0 (0.0)	0 (0.0)	242 (45.3)	
**Birth details**				
Multiple birth				0.83
1	479 (97.2)	165 (97.1)	515 (96.4)	
2	14 (2.8)	5 (2.9)	18 (3.4)	
≥3	0 (0.0)	0 (0.0)	1 (0.2)	
Birth weight (g), mean (SD)	3023.8 (707.2)	3283.2 (546.5)	3191.8 (610.3)	<0.001
Birth height (cm), mean (SD)	47.9 (4.5)	49.1 (2.8)	49.0 (3.1)	<0.001
Gestational age (weeks), mean (SD)	38.7 (2.5)	39.1 (1.6)	39.0 (1.9)	0.011
**Congenital diseases & malformations**				
Chromosomal anomalies	19 (3.9)	1 (0.6)	21 (3.9)	0.090
Congenital malformations of the respiratory system	39 (7.9)	2 (1.2)	35 (6.6)	0.008
Cystic fibrosis	0 (0.0)	0 (0.0)	0 (0.0)	
Congenital malformations of the heart	83 (16.8)	5 (2.9)	51 (9.6)	<0.001
Congenital malformations of the vascular system	46 (9.3)	3 (1.8)	22 (4.1)	<0.001
Other congenital malformations of the digestive system	61 (12.4)	4 (2.4)	25 (4.7)	<0.001
Metabolic disorders	3 (0.6)	0 (0.0)	0 (0.0)	0.12

© 2024 Laager R et al. *JAMA Network Open.*

Characteristic	Cleft palate only, n (%)	Cleft lip only, n (%)	Cleft lip and palate, n (%)	P-value
Congenital malformations of the urinary system	35 (7.1)	4 (2.4)	15 (2.8)	0.001
Congenital malformations of genital organs	30 (6.1)	7 (4.1)	21 (3.9)	0.25
Congenital malformations and deformations of the musculoskeletal system	111 (22.5)	9 (5.3)	55 (10.3)	<0.001
Other congenital malformations	144 (29.2)	2 (1.2)	43 (8.1)	<0.001
Syndromes combined	233 (47.3)	26 (15.3)	150 (28.1)	<0.001
**Neonatological issues**				
Intrauterine hypoxia	25 (5.1)	2 (1.2)	26 (4.9)	0.083
Birth asphyxia	25 (5.1)	1 (0.6)	16 (3.0)	0.016
Adaptive dysfunction	42 (8.5)	7 (4.1)	27 (5.1)	0.033
Neonatal respiratory issues	79 (16.0)	8 (4.7)	42 (7.9)	<0.001
Respiratory distress of newborn	124 (25.2)	19 (11.2)	79 (14.8)	<0.001
Neonatal cardiovaskular issues	58 (11.8)	5 (2.9)	36 (6.7)	<0.001
Neonatal haemorrhages	11 (2.2)	1 (0.6)	4 (0.7)	0.078
Neonatal haemolysis	4 (0.8)	1 (0.6)	2 (0.4)	0.66
Other perinatal haematological disorders	35 (7.1)	1 (0.6)	11 (2.1)	<0.001
Neonatal transitory endocrine & metabolic disorders	61 (12.4)	8 (4.7)	35 (6.6)	<0.001
Neonatal digestive system disorders	26 (5.3)	0 (0.0)	7 (1.3)	<0.001
Neonatal temperature regulation and integument conditions	22 (4.5)	5 (2.9)	14 (2.6)	0.25
Neurological disorders of fetus and newborn	30 (6.1)	0 (0.0)	15 (2.8)	<0.001
Death in Birth Hosp	11 (2.2)	1 (0.6)	10 (1.9)	0.39
Resuscitation	46 (9.3)	8 (4.7)	27 (5.1)	0.013
Intubation	55 (11.2)	2 (1.2)	23 (4.3)	<0.001

Abbreviation: SD, standard deviation.

© 2024 Laager R et al. *JAMA Network Open.*

Table S3 – Baseline characteristics with or without surgical cleft repair

Characteristic	No cleft repair, n (%)	Cleft repair, n (%)	P-value
**N**	N=453	N=744	
**Sex**			0.46
Female	194 (42.8)	335 (45.0)	
Male	259 (57.2)	409 (55.0)	
**Type of surgery**			<0.001
None	453 (100.0)	0 (0.0)	
Lip repair only	0 (0.0)	211 (28.4)	
Palate repair only	0 (0.0)	291 (39.1)	
Lip and palate repair	0 (0.0)	242 (32.5)	
**Birth details**			
Multiple birth			0.35
1	440 (97.1)	719 (96.6)	
2	12 (2.6)	25 (3.4)	
≥3	1 (0.2)	0 (0.0)	
Birth weight (g), mean (SD)	3020.4 (717.5)	3205.8 (596.4)	<0.001
Birth height (cm), mean (SD)	48.2 (4.1)	48.8 (3.6)	0.007
Gestational age (weeks), mean (SD)	38.7 (2.4)	39.0 (2.0)	0.009
**Congenital diseases & malformations**			
Chromosomal anomalies	21 (4.6)	20 (2.7)	0.072
Congenital malformations of the respiratory system	32 (7.1)	44 (5.9)	0.43
Cystic fibrosis	0 (0.0)	0 (0.0)	
Congenital malformations of the heart	72 (15.9)	67 (9.0)	<0.001
Congenital malformations of the vascular system	47 (10.4)	24 (3.2)	<0.001
Other congenital malformations of the digestive system	45 (9.9)	45 (6.0)	0.013
Metabolic disorders	2 (0.4)	1 (0.1)	0.30

© 2024 Laager R et al. *JAMA Network Open.*

Characteristic	No cleft repair, n (%)	Cleft repair, n (%)	P-value
Congenital malformations of the urinary system	33 (7.3)	21 (2.8)	<0.001
Congenital malformations of genital organs	28 (6.2)	30 (4.0)	0.093
Congenital malformations and deformations of the musculoskeletal system	95 (21.0)	80 (10.8)	<0.001
Other congenital malformations	89 (19.6)	100 (13.4)	0.004
Syndromes combined	165 (36.4)	244 (32.8)	0.20
**Neonatological issues**			
Intrauterine hypoxia	23 (5.1)	30 (4.0)	0.39
Birth asphyxia	24 (5.3)	18 (2.4)	0.009
Adaptive dysfunction	36 (7.9)	40 (5.4)	0.077
Neonatal respiratory issues	62 (13.7)	67 (9.0)	0.011
Respiratory distress of newborn	109 (24.1)	113 (15.2)	<0.001
Neonatal cardiovaskular issues	49 (10.8)	50 (6.7)	0.013
Neonatal haemorrhages	8 (1.8)	8 (1.1)	0.31
Neonatal haemolysis	5 (1.1)	2 (0.3)	0.066
Other perinatal haematological disorders	34 (7.5)	13 (1.7)	<0.001
Neonatal transitory endocrine & metabolic disorders	50 (11.0)	54 (7.3)	0.024
Neonatal digestive system disorders	19 (4.2)	14 (1.9)	0.018
Neonatal temperature regulation and integument conditions	26 (5.7)	15 (2.0)	<0.001
Neurological disorders of fetus and newborn	26 (5.7)	19 (2.6)	0.005
Death in Birth Hosp	22 (4.9)	0 (0.0)	<0.001
Resuscitation	41 (9.1)	40 (5.4)	0.014
Intubation	52 (11.5)	28 (3.8)	<0.001

Abbreviation: SD, standard deviation.

© 2024 Laager R et al. *JAMA Network Open.*

Table S4 – Incidence rate ratios (IRR) for hospitalizations considering any diagnosis of airway infection (primary or secondary diagnosis)

	N (%) Controls	N (%) Clefts	IRR	95% CI	P-value
**Overall**	45717 (5.3)	166 (13.9)	3.44	3.05 to 3.89	<0.001
**Pre-surgery**	27366 (3.2)	84 (11.3)	4.15	3.44 to 5.01	<0.001
**Post-surgery**	20683 (2.4)	36 (4.8)	2.41	1.83 to 3.16	<0.001

Abbreviations: CI, confidence interval; IRR, incidence rate ratio

© 2024 Laager R et al. *JAMA Network Open.*

Figure S1 - Distribution of surgical modalities

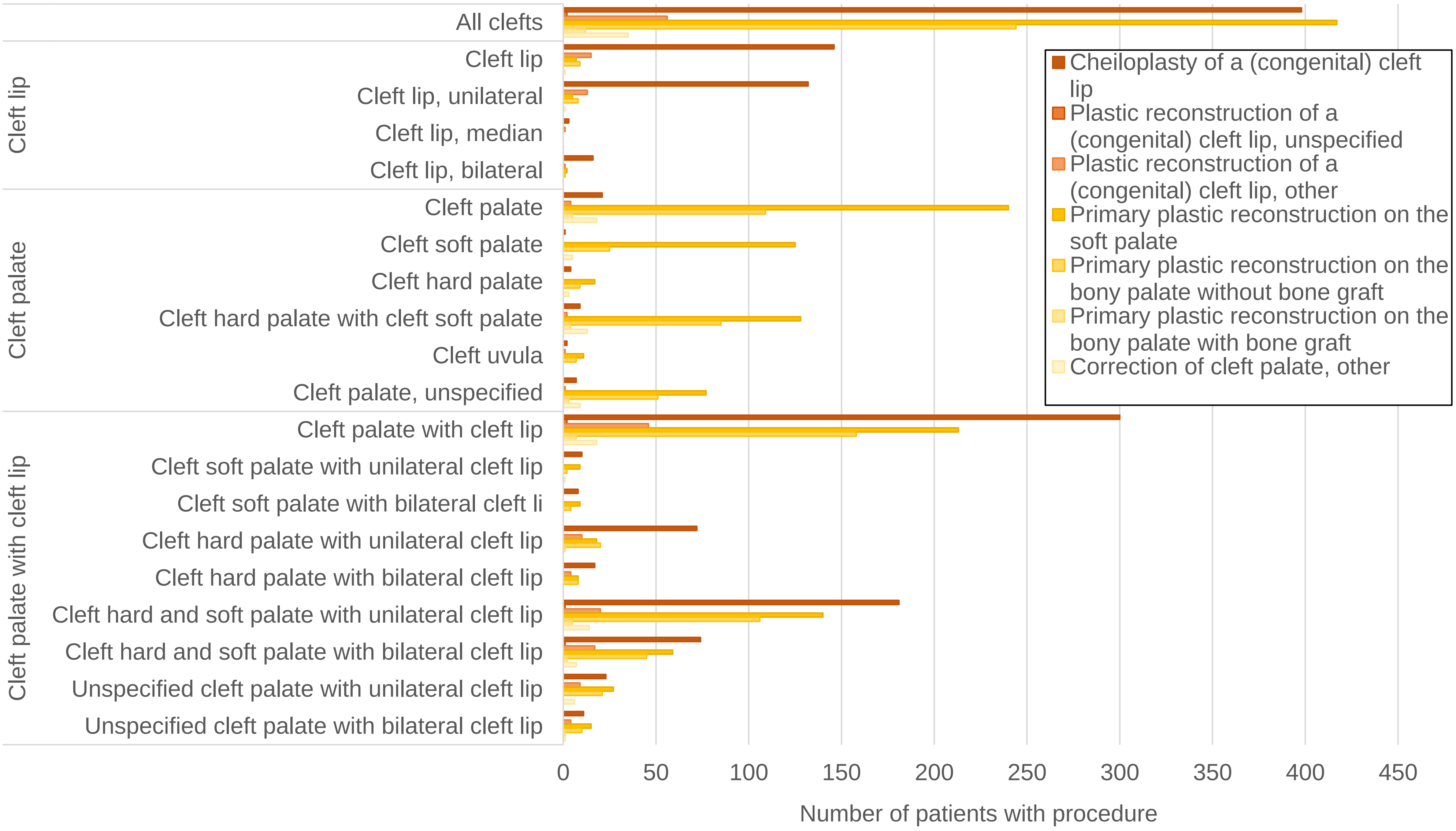

© 2024 Laager R et al. *JAMA Network Open.*

Figure S2 – Distribution of surgical cleft repair by age

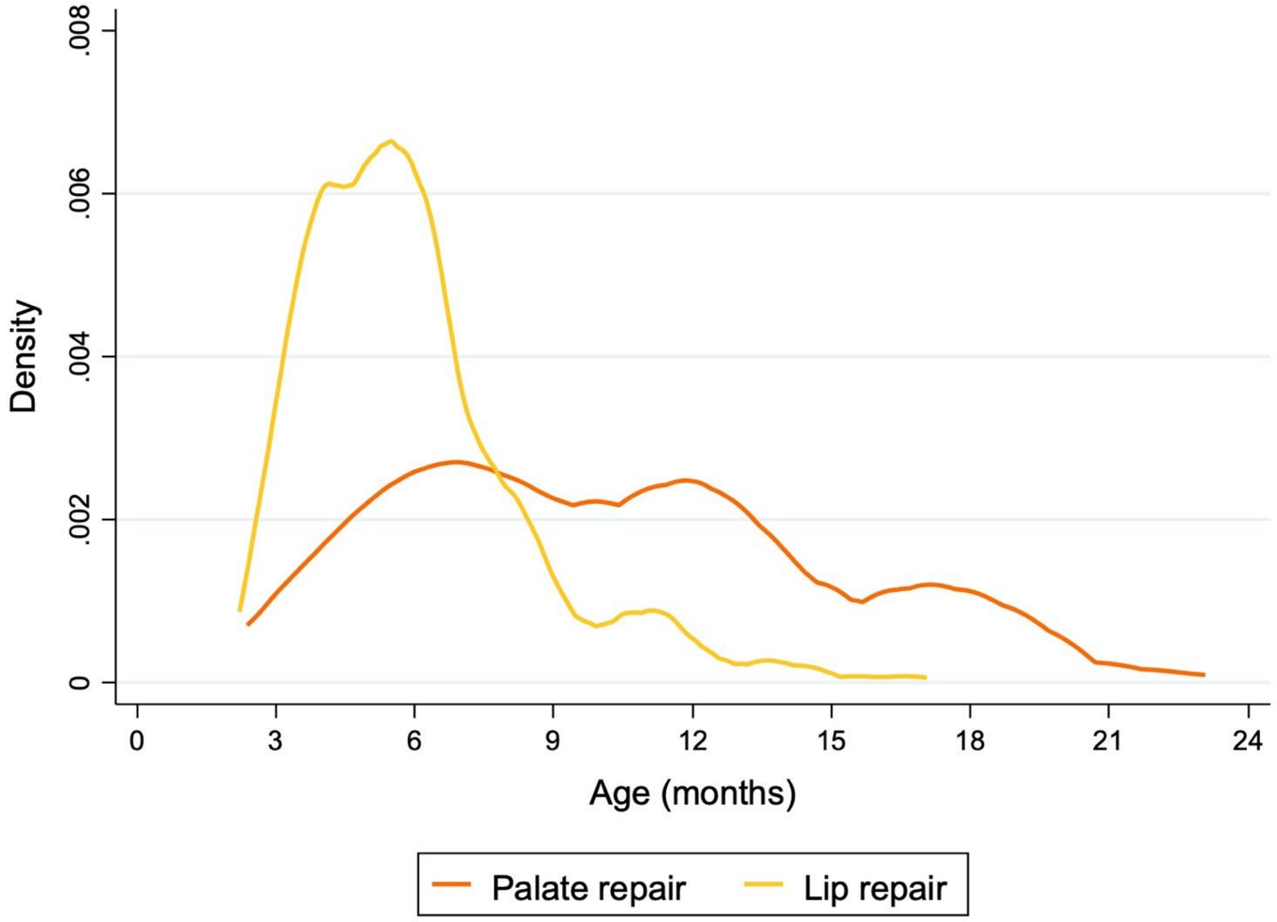

© 2024 Laager R et al. *JAMA Network Open.*

Figure S3 – Hospitalization rates before and after first cleft repair

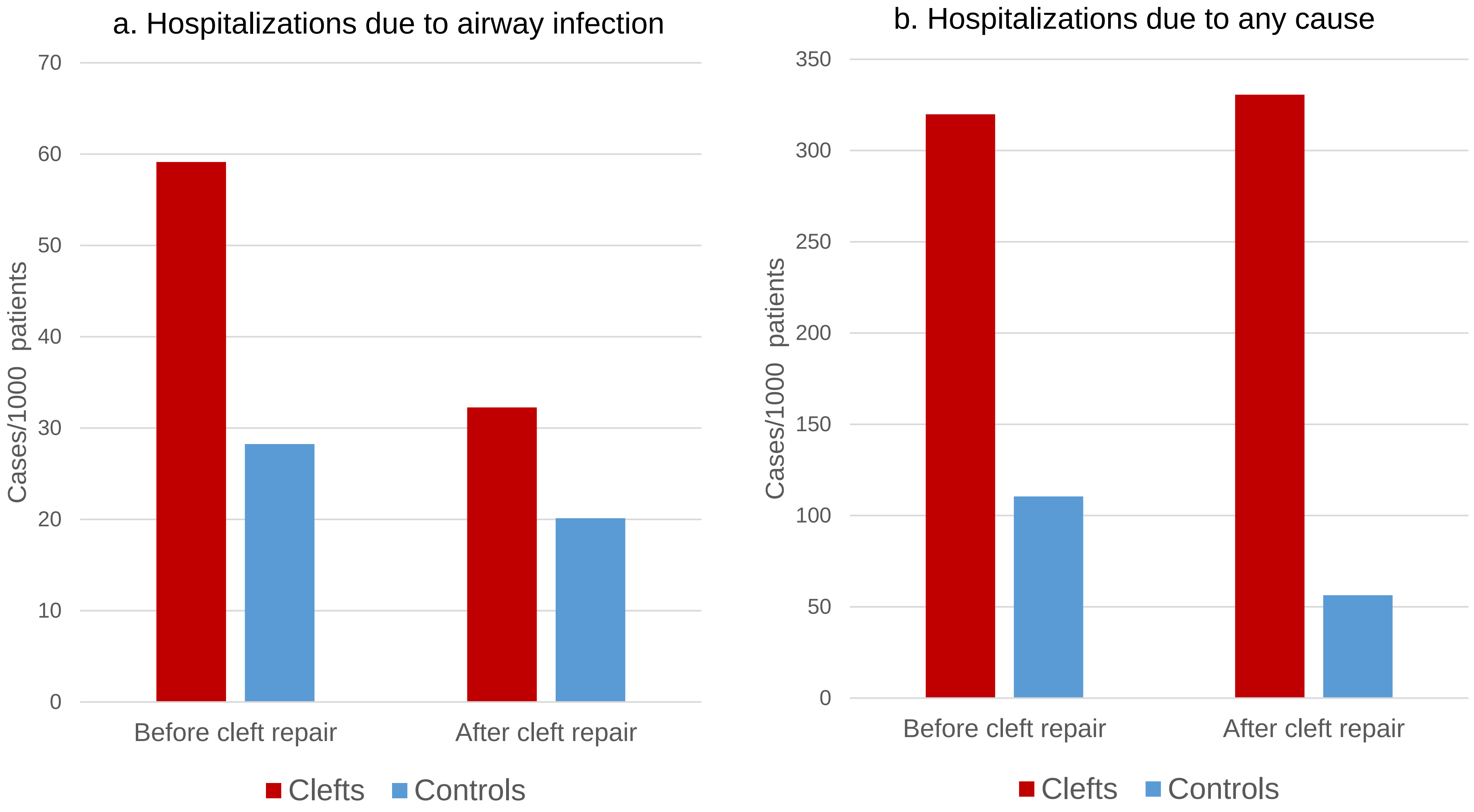

© 2024 Laager R et al. *JAMA Network Open.*

Figure S4 - Hospitalization rates before and after first lip repair

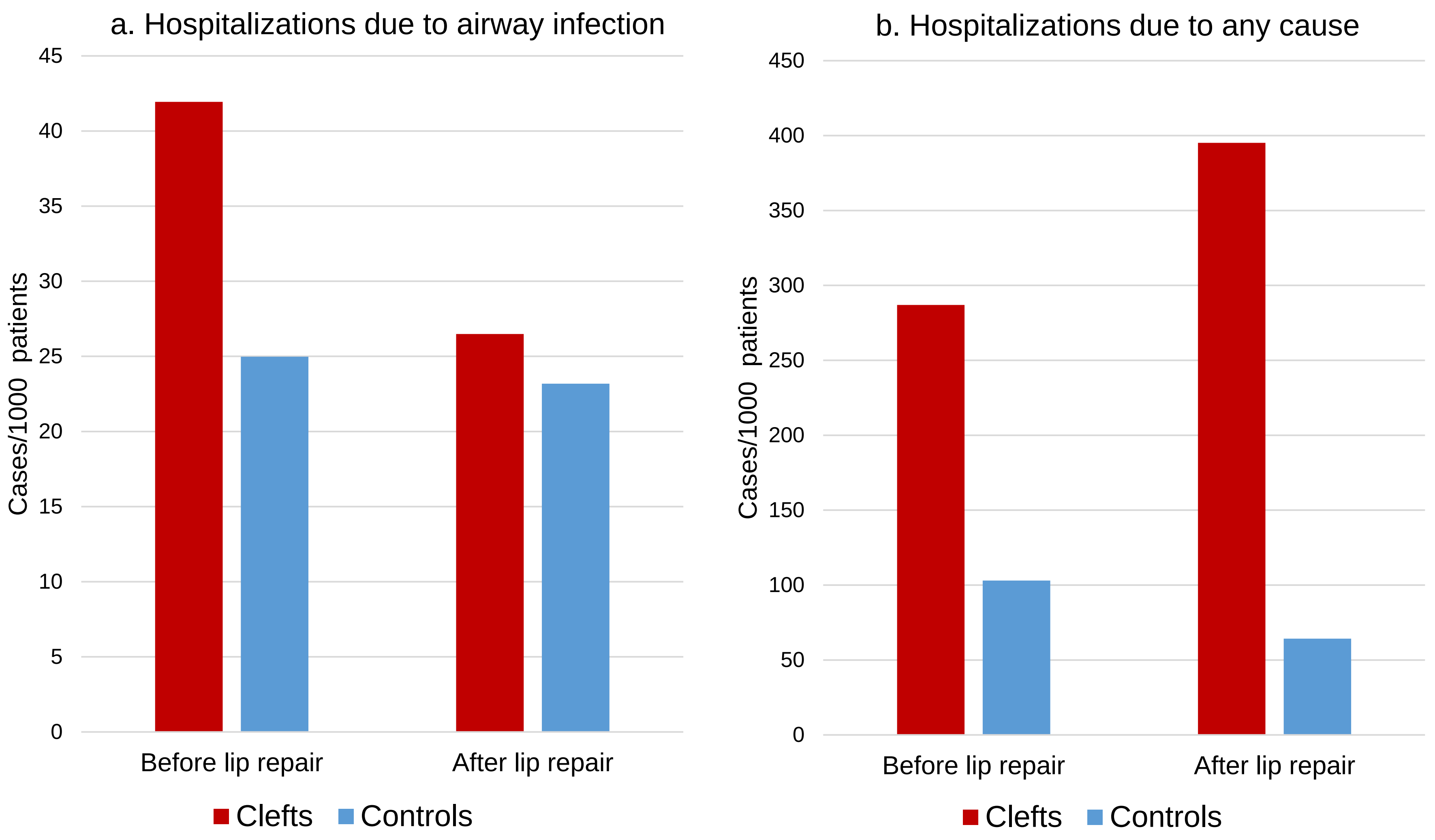

© 2024 Laager R et al. *JAMA Network Open.*

Figure S5 – In-hospital outcomes before and after surgical li repair

Outcome	Pre-surgery OR (95% CI)	Pre-surgery P-value	Post-surgery OR (95% CI)	Post-surgery P-value
**Respiratory support**	0.84 (0.58 - 1.23)	=0.38	0.68 (0.36 - 1.28)	=0.23
**Severe adverse events**	0.88 (0.59 - 1.30)	=0.52	1.37 (0.68 - 2.77)	=0.38
**ECMO**	8.77 (3.92 - 19.63)	<0.001	0.73 (0.10 - 5.20)	=0.75
**Intubation**	1.31 (0.85 - 2.00)	=0.22	1.49 (0.74 - 3.03)	=0.26
**Resuscitation**	0.32 (0.13 - 0.77)	=0.011	1.70 (0.24 - 12.21)	=0.60
**In-hospital death**	n/a	n/a	1.88 (0.26 - 13.45)	=0.53
**Overall mortality**	n/a	n/a	7.05 (0.99 - 50.33)	=0.05

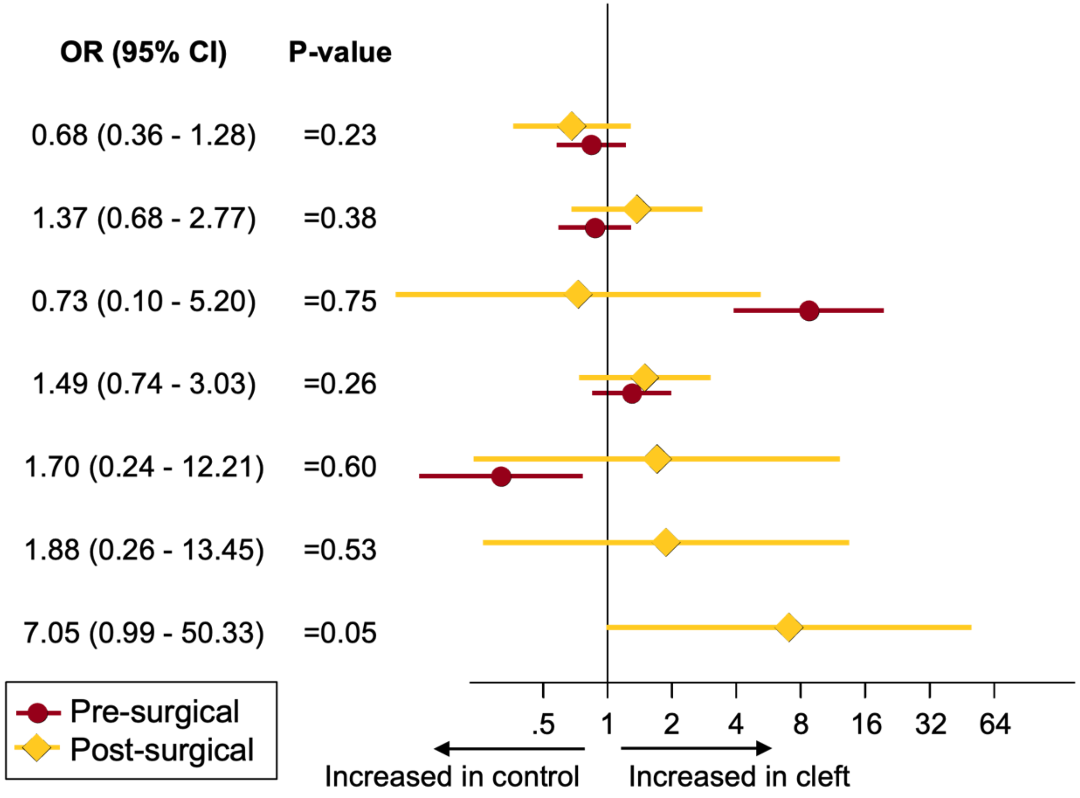

Graphical depiction of odds ratios (OR) as forest plots with corresponding 95% CI and p-values on a natural logarithmic scale.
Abbreviations: CI, confidence interval; OR, odds ratio; ECMO, extracorporeal membrane oxygenation

© 2024 Laager R et al. *JAMA Network Open.*

Figure S6 - Hospitalization rates before and after first palate repair

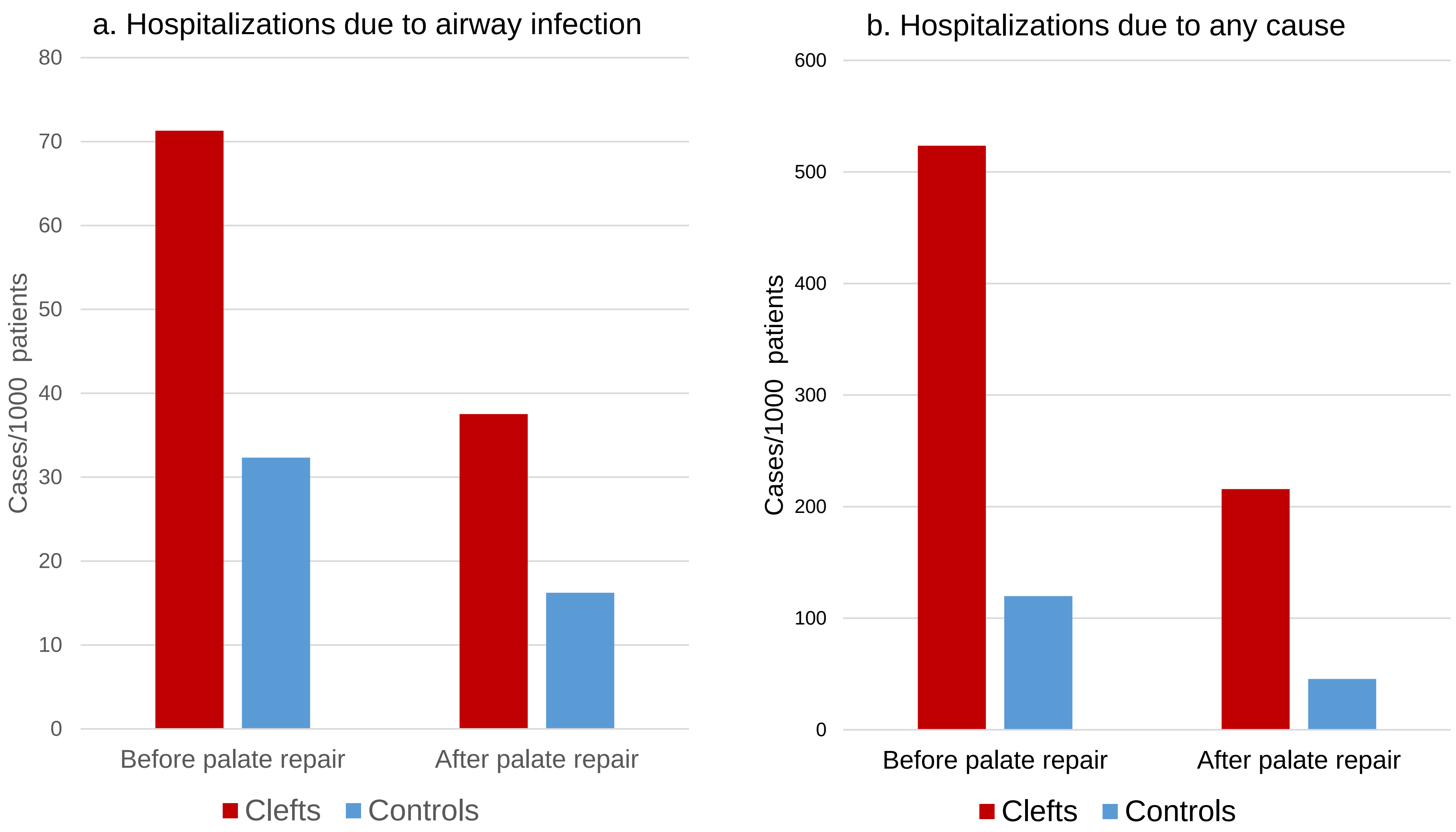

© 2024 Laager R et al. *JAMA Network Open.*

Figure S7 – In-hospital outcomes before and after surgical palate repair

Outcome	Pre-surgery OR (95% CI)	Pre-surgery P-value	Post-surgery OR (95% CI)	Post-surgery P-value
**Respiratory support**	1.84 (1.49 - 2.27)	<0.001	0.93 (0.46 - 1.90)	=0.85
**Severe adverse events**	1.59 (1.25 - 2.03)	<0.001	1.41 (0.58 - 3.45)	=0.45
**ECMO**	7.80 (4.18 - 14.58)	<0.001	1.72 (0.24 - 12.37)	=0.59
**Intubation**	2.67 (2.09 - 3.40)	<0.001	1.54 (0.63 - 3.76)	=0.34
**Resuscitation**	0.49 (0.28 - 0.87)	=0.02	1.00 (0.00 - 0.00)	<0.001
**In-hospital death**	n/a	n/a	3.37 (0.47 - 24.34)	=0.23
**Overall mortality**	n/a	n/a	10.88 (1.52 - 77.88)	=0.02

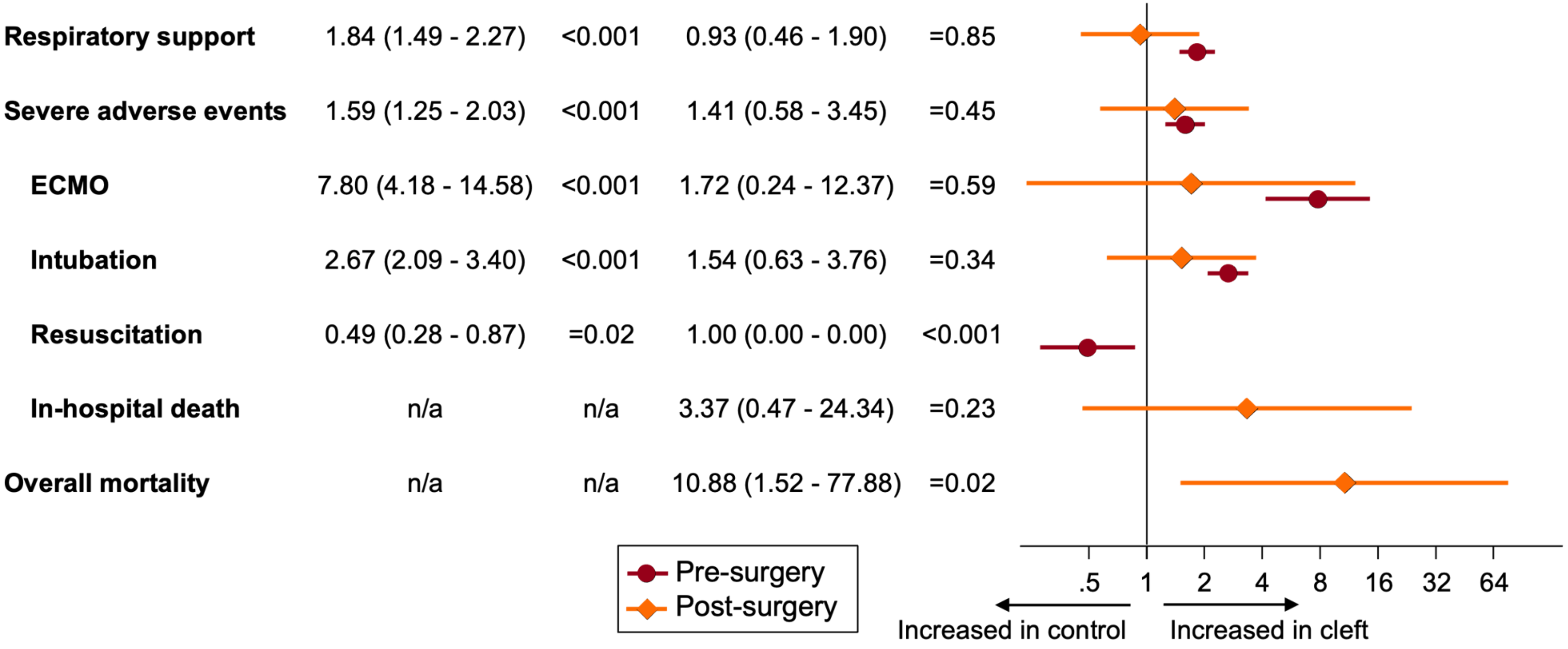

Graphical depiction of odds ratios (OR) as forest plots with corresponding 95% CI and p-values on a natural logarithmic scale.
Abbreviations: CI, confidence interval; OR, odds ratio; ECMO, extracorporeal membrane oxygenation

© 2024 Laager R et al. *JAMA Network Open.*

Figure S8 – Monthly incidence rates of hospitalizations considering any diagnosis of airway infection (primary or secondary diagnosis)

a. Hospitalizations considering any diagnosis of airway infection

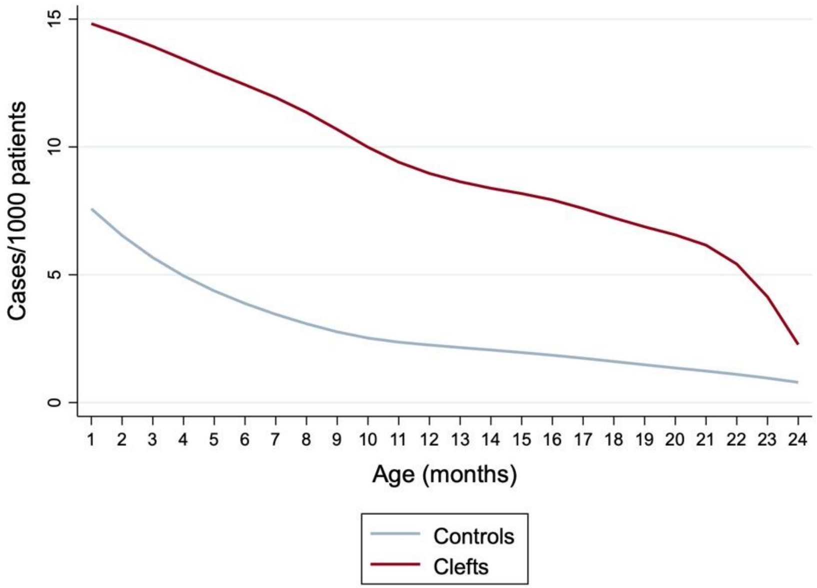

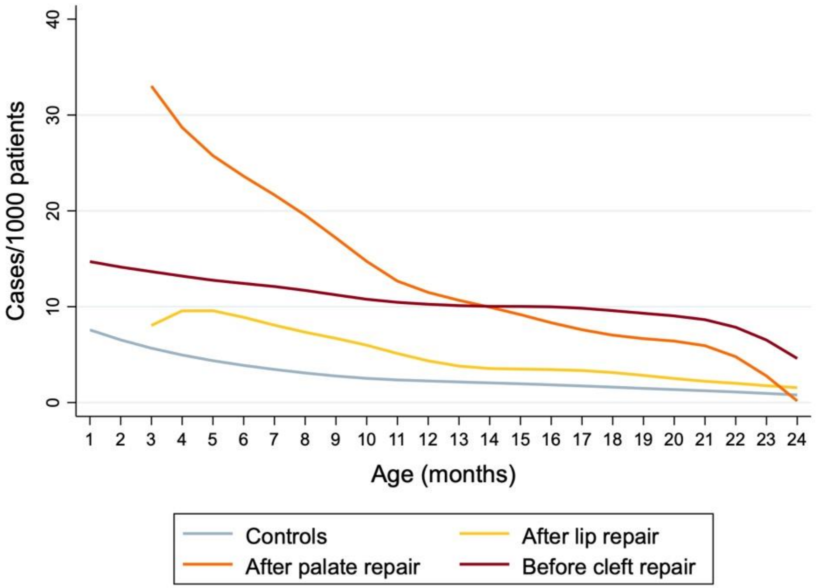

© 2024 Laager R et al. *JAMA Network Open.*
