# Supplementary material for: Hospitalization Trends for Airway Infections and In-Hospital Complications in Cleft Lip and Palate
Source: JAMA Netw Open. 2024 Sep 12;7(9):e2428077. doi: 10.1001/jamanetworkopen.2024.28077 (PMC11393727; doi:10.1001/jamanetworkopen.2024.28077)
Supplement: Supplement 2. — Data Sharing Statement [file jamanetwopen-e2428077-s002.pdf]

Data Sharing Statement

Laager. Hospitalization Trends for Airway Infections and In-Hospital Complications in Cleft Lip and Palate. *JAMA Netw Open*. Published September 12, 2024.
doi:10.1001/jamanetworkopen.2024.28077

Data
Data available: No

Additional Information
Explanation for why data not available: The data that support the findings of this study are available upon request from the Swiss Federal Statistical Office (Neuchâtel, Switzerland). Restrictions apply to the availability of these data, which were used under license for this study. Data are available as part of the data on “Medizinische Statistik der Krankenhäuser” with the permission of the Swiss Federal Statistical Office, Section Health Services and Population Health.
